# Evaluation of the safety and effectiveness of tubal inflammatory drugs in patients with incomplete tubal obstruction after four-dimensional hysterosalpingo-contrast-sonography examination

**DOI:** 10.1186/s12884-022-04722-y

**Published:** 2022-05-07

**Authors:** Yue Zhang, Qin Wang, Chun-yan Gao, Hong-ju Tian, Wen-jiao He, Xi Zhang, Xi Xiong

**Affiliations:** 1 Department of Obstetrics and Gynecology, the Second Affiliated Hospital of Army Medical University, Chongqing, 400037 PR China; 2 Department of Radiology, the Second Affiliated Hospital of Army Medical University, Chongqing, 400037 PR China

**Keywords:** Tubal inflammatory drugs, Incomplete tubal obstruction, Four-dimensional hysterosalpingo-contrast-sonography (4D-HyCoSy)

## Abstract

**Background:**

To investigate the safety and effectiveness of tubal inflammatory drugs in patients with incomplete tubal obstruction of at least one side after four-dimensional hysterosalpingo-contrast-sonography (4D-HyCoSy) examination.

**Methods:**

Two hundred fifteen cases of tubal incomplete obstruction were diagnosed by ultrasonography from February 2019 to November 2020.According to retrospective analysis,the patients in this study were divided into experimental and control groups; the experimental group combined with salpingitis drugs, and the control group received blank control. Basic information, degree of pain, postoperative complications, and pregnancy rate were then compared between the two groups.

**Results:**

Compared with the control group, there was no significant difference in the basic information; in preoperative, intraoperative, or postoperative pain; or in postoperative complications (*P* > 0.05). The cumulative pregnancy rate of the experimental group (26.8%) was statistically different from that of the control group (14.4%) (*P* < 0.05).

**Conclusions:**

We observed that for infertile patients with incomplete obstruction of at least one fallopian tube as diagnosed by contrast-enhanced ultrasonography, salpingitis-treatment drugs effectively improved the pregnancy rate postoperatively, with high effectiveness and safety. This regimen is thus worthy of further investigation and promotion in the future.

## Background

Infertility is a common reproductive health issue that is defined as the inability to conceive for 12 months or more during unprotected sexual intercourse [1]. Many studies reported that more couples suffer from infertility, which now accounts for 5–15% of couples of reproductive age. About one third of infertility is related to female factors [2], while tubal infertility accounts for 25–35% of female infertility [3, 4]. The primary cause of tubal infertility is salpingitis [5, 6], which is mainly caused by various forms of inflammation of the female reproductive tract—including chronic pelvic inflammation, induced abortion, and endometriosis [7, 8]. Complete tubal obstruction caused by salpingitis directly leads to infertility, while incomplete tubal obstruction can cause infertility and ectopic pregnancy [9]. Salpingitis is the first causative factor of tubal pregnancy which accounts for more than 90% of ectopic pregnancies [10]. Thus, incomplete tubal obstruction is considered to be more harmful, and we therefore need to seek better treatment for patients with incomplete tubal obstruction.

Therapeutic methods for tubal infertility include pelvic surgery, laparoscopic tubal hydrotubation [11], tubal hydrotubation, and radiation-mediated tubal intervention. Pelvic surgery (because of its great trauma and exposure of the fallopian tubes to air) increases the risk of oxidative adhesions, and is prone to pelvic and fallopian tube re-adhesion after surgery; it has therefore been used less frequently [12]. Although laparoscopic hydrotubation for tubal can display the fallopian tube morphology, dissolve the adhesions between the fallopian tubes and surrounding tissues, allow experimentation of the process of Meilan outflow through the tubal, and provide accurate determination of the location of tubal obstruction [13], its operative time is overly long, expensive, and carries the risk of surgical trauma and anesthesia. It is therefore not the preferred treatment for tubal obstruction. Interventional radiologist’s approach to fallopian tube recanalization refers to the placement of a super-slip guidewire to separate and bond the obstructed fallopian tube by lipiodol angiography under X-ray monitoring. This method is simple, less traumatic, and has a good curative effect. Dr. Han Zhigang of the Affiliated Hospital of Obstetrics and Gynecology of Fudan University reported a recanalization rate of 63.9% in 459 patients. After 1 1/2 years of follow-up, the intrauterine pregnancy rate was 43.9%, and the ectopic pregnancy rate was 2.1%. Although this method exhibits great effectiveness, both physicians and patients undergo radiologic exposure, potential iodine reactions, and a long preparation time during operation—which also reflect invasive treatment. Therefore, at present, the clinical treatment is principally based on tubal hydrotubation, which was first proposed by Dr. Hideo Yagi in 1929, and is a novel diagnostic technique that enables one to judge whether the oviduct is unobstructed or not without the need for air and carbon dioxide [14]. The treatment of tubal obstruction by tubal hydrotubation uses the static pressure of liquid through the tubal lumen to separate the luminal adhesion band and mucus plug so as to achieve tubal recanalization. In 1947, Rubin published a report that tubal infertility patients can become pregnant soon after receiving tubal hydrotubation, with a pregnancy rate after gas injection of 18% and a pregnancy rate after hysterosalpingo hydrotubation of 25–40% [15, 16]. In 1966, Salomy et al. [17] indicated that a mixture of hydrocortisone, streptomycin, and chymotrypsin can enhance the dredging effect of tubal hydrotubation in the treatment of patients with tubal obstruction. Zou et al. at the Traditional Chinese Medicine Hospital of Jilin Province reported a therapeutic effect that attained 93.3% with respect to incomplete oviduct obstruction using tubal hydrotubation with 40,000 units of Gentamicin injection, 5 mg of dexamethasone injection, 4000 units of chymotrypsin, and 20 mL of water for injection [18].

Hydrotubation as a test method to judge whether the oviduct is unobstructed or not entails a certain subjectivity, and it is very difficult to understand the morphology and the obstruction site of the oviduct. With the development of diagnosis and treatment technologies, 4D-HyCoSy has replaced tubal hydrotubation. Real-time monitoring by ultrasonography allows the visual display of the shape and obstruction site of the oviduct and to diagnose tubal obstruction, and its accuracy is in good agreement with that of laparoscopic salpingeal methylene-blue staining hydrotubation [19]. On this basis, we have therefore offered the hypothesis that after completion of ultrasonographic hysterosalpingography—and for those patients with at least 1 fallopian tube diagnosed with incomplete obstruction—we continue intrauterine injection of drugs to treat tubal inflammation, consolidate the dredging effect of contrast media on the fallopian tube after salpingography, improve the drug concentration at the lesion site, and further treat the tubal structural anomalies caused by inflammation. This allowed for functional changes and improved the pregnancy rate after surgery.

## Materials and methods

This retrospective study was approved by the Medical Ethics Committee of Second Affiliated Hospital of Army Medical University (2018-YD-010-01).

In accordance with *World Medical Association Declaration of Helsinki: Ethical Principles for Medical Research Involving Human Subjects*, 2013, all study participants gave written informed consent regarding study procedures and treatment modalities after the procedures had been fully explained to them.

All the records of the 215 patients who accepted this project in our center between February 2019 and November 2020 were extracted through in-depth analysis. These patients participated in the study and were followed up for at least 6 months postoperatively.

Inclusion criteria were as follows: (1) Sex was forbidden after a menstrual cycle, HyCoSy was performed 3–7 days after clean menstruation, and the diagnosis was incomplete obstruction of at least one fallopian tube; (2) men had normal semen; (3) there were no endocrine diseases (such as hyperprolactinemia or pituitary diseases); and (4) there was no uterine deformity.

Exclusion criteria were (1) not meeting the inclusion criteria above; (2) contrast media reflux occurring during contrast; (3) the presence of tuberculous salpingitis, or acute or subacute pelvic inflammatory disease; and (4) allergy to contrast media, gentamicin, dexamethasone, or chymotrypsin.

### Operating steps

Atropine (0.5 mg) was injected intramuscularly 30 minutes before examination to prevent pseudo-obstruction caused by hysterosalpingeal spasm.

We asked the patient to empty her bladder and take the lithotomy position, and we disinfected, spread towels, placed a No. 12 Foley catheter into the uterine cavity, and injected 0.8 to 1.2 mL of saline into the balloon.

Fifty milligrams of SonoVue (SonoVue, Bracco S.p.A., Milan, Italy) contrast agent was completely dissolved in 5 mL of 0.9% saline, and 2.4 mL was taken and diluted in 17.6 mL of 0.9% saline for HyCoSy.

HyCoSy was performed and the degree of pain, vagal reflex, resistance to contrast injection, and the presence or absence of reflux were recorded.

According to the HyCoSy results, patients with incomplete tubal obstruction on at least one side were divided into an experimental group and a control group. The observation group injected 20 mL of drug mixture (40,000 U of chymotrypsin + 80,000 u of gentamycin + 5 mg of dexamethasone + normal saline) into the balloon catheter by TVS observing the flow of the mixture of therapeutic drugs in the fallopian tube to determine the drug into the fallopian tube incomplete obstruction side; it entered into the fallopian tube through the uterine cavity to reach the slight adhesions or stenosis of the fallopian tube. At the same time, the degree of pain, vagal reflex, resistance to injection, reflux, and postoperative bleeding were recorded. The control group, as a blank control, only completed HyCoSy.

Image analysis was performed by 2 obstetrics and gynecology ultrasonographic physicians (intermediate or above, working for no less than 5 years).

The evaluation criteria for incomplete tubal obstruction were as follows.

**Fig. 1:**
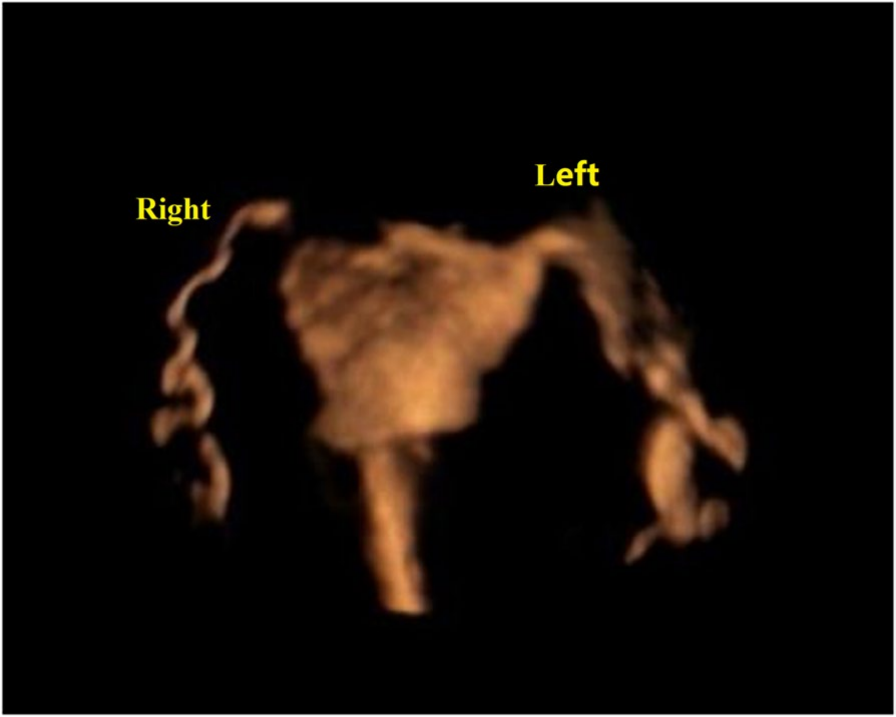
Ultrasonography showing nodular thickening or a walking curl or inversion in the local part of the fallopian tube

Ultrasonography showed nodular thickening or a walking curl or inversion in the local part of the fallopian tube, a small amount of contrast agent at the umbrella end spilled in a line or a sheet shape, a semicircular strong echo band around the ovary, and limited diffusion of contrast agent in the pelvis (Fig. 1). When the contrast medium was injected, there was resistance and reflux. The patient’s pain was tolerable, and the uterine cavity’s resistance was mild [13, 20].

Pain was quantified using the Visual Analogue Scale (VAS, 0–10 points), with 0 indicating no pain at all and 10 indicating severe pain. Pain was divided into 4 grades: 0 for no pain, 1–3 for mild, 4–6 for moderate, and 7–10 for severe pain. The amount of bleeding and the degree of pain were recorded [21, 22].

After the examination, the experimental group and the control group were followed for at least 6 months, and we noted the mode of pregnancy and pregnancy rate. We analyzed all data obtained from the cases with SPSS 26.0, using the *t* test for continuous variables and Chi-squared test for categorical variables. Values with a *P* < 0.05 were considered statistically significant.

## Results

During the study period,215 patients participated, of which 22 were lost to follow up (i.e., patients could not be contacted after several telephone calls). Among these, there were 15 cases in the experimental group and 7 cases in the control group who failed to provide relevant information such as late complications and pregnancy after completing the HyCoSy. operation, and were thus excluded from the study. Complete information was obtained in 193 patients, and late complications and pregnancy information were obtained by telephone and re-visit.

There were no significant differences in age, body mass index, duration of pregnancy preparation, or type of infertility between the 2 groups (Table 1).

**Table 1:** Basic information on subjects

Classification	E- group (***n*** = 82)	C-group (***n*** = 111)	P
**Age (year)**	28.75 ± 4.09 (21 ~ 40)	30.58 ± 4.52 (21 ~ 41)	0.247
**BMI (kg/m**^**2**^**)**	21.75 ± 3.22 (15.79 ~ 34.63)	22.13 ± 3.16 (16.44 ~ 35.25)	0.889
**Duration of subfertility**			
Months	20.37 ± 15.55 (2 ~ 108)	23.72 ± 25.09(0 ~ 204)	0.081
**Dysmenorrhea (n/%)**	47 (51.22%)	49 (44.14%)	0.07
**Types of infertility**			0.072
Primary infertility (n/%)	37 (45.12%)	36(32.43%)	
Secondary infertility (n/%)	45 (54.88%)	75(67.57%)	
**Menstrual cycle (days)**	30.53 ± 6.70 (20 ~ 75)	30.22 ± 5.04 (22 ~ 50)	0.376
**Menstrual period (days)**	5.81 ± 1.50 (2 ~ 11)	5.75 ± 1.35 (3 ~ 8)	0.832
**Ectopic pregnancy (n/%)**	7 (8.54%)	13 (11.71%)	0.474
**Endometrial thickness (mm)**	6.66 ± 1.83	6.49 ± 2.13	0.068

Among the 82 patients in the experimental group, 1 had a tubal pregnancy,and 22 had intrauterine pregnancies (4 induced abortion); and the cumulative intrauterine pregnancy rate was 9.7% 1 month and 26.8% 6 months after the operation.

Of the 111 patients in the control group, 1 had a tubal pregnancy,and 16 had intrauterine pregnancies (3 had abortion). The cumulative intrauterine pregnancy rate was 2.7% 1 month and 14.4% 6 months after the operation.

Between the two groups of data, the 6-month cumulative intrauterine pregnancy rate was statistically significant (*p* = 0.032).

Of the 193 patients, we observed in the 82 of the experimental group,1 case of mild vaso-vagal reflex, 1 case of severe vagal reflex, 68 cases of mild pain, 12 cases of moderate pain, and 0 cases of severe pain after uterine-catheter insertion, during ultrasound contrast-agent injection, and during salpingitis drug injection, respectively. Fifty cases had minimal vaginal bleeding after treatment none had bleeding greater than menstrual volume; 43 had bleeding within 3 days and the rest did not exceed 7 days. One case had fever and other patients had no fever, pelvic inflammation or other infectious symptoms. In the 111 cases of the control group, 5 cases had mild vaso-vagal reflex, 2 cases had severe vagal reflex. Eighty-four cases had mild pain after insertion of the uterine catheter, during injection of ultrasound contrast agent, and during injection of salpingitis drug respectively. These were 25 cases with moderate pain and 0 cases with severe pain. Fifty-eight cases showed vaginal bleeding after treatment and reduced menstrual volume, with 52 cases showing < 3 days of bleeding, with the remainder exhibiting 3–7 days of bleeding. We observed no fever, pelvic inflammation, or other infectious symptoms in any patient.

There was no significant difference in VAS scores between the experimental group and the control group in terms of preoperative catheterization, intraoperative microbubble angiography, injection of salpingitis drugs, or pain 30 minutes after surgery (*P* > 0.05) (Table 2).

**Table 2:** Visual analogue scale (VAS) scores and postoperative adverse reactions

Classification	E- group (***n*** = 82)	C- group (***n*** = 111)	P
**VAS scores**			
Implantation of balloon catheter into uterine cavity	1.89 ± 0.86	1.99 ± 1.11	0.161
Tubal injection of Microbubble contrast agent	2.27 ± 1.10	2.85 ± 1.12	0.485
Half an hour after operation	0.17 ± 0.44	0.21 ± 0.55	0.248
**Mild vaso-vagal reactions (pallor, nausea, sudation, hypotension, bradycardia)**	1/82 (1.23%)	5/111 (4.50%)	0.194
Implantation of balloon catheter into uterine cavity	0/82 (0%)	3/111 (2.70%)	0.134
Tubal injection of Microbubble contrast agent	1/82 (1.23%)	0/111 (0%)	0.243
Half an hour after operation	0/82 (0%)	2/111 (1.80%)	0.222
**Severe vaso-vagal reactions (vomiting,confusion, syncope)**	1/82 (1.23%)	2/111 (1.80%)	0.747
Implantation of balloon catheter into uterine cavity	0/82 (0%)	1/111 (0.90%)	0.389
**postoperative hemorrhage**	50/82 (60.98%)	58/111 (52.25%)	0.228

## Discussion

Infertility caused by tubal factors is still a common cause of female infertility [23], which is mainly tubal inflammation or pelvic inflammation caused by various reasons. The primary pathologic mechanism underlying incomplete tubal obstruction is tubal mucositis caused by inflammation—which causes tubal mucosal swelling, interstitial hyperemia and edema, and necrosis or shedding of tubal epithelial cells—thus leading to tubal lumen narrowing, slight adhesion, or partial atresia at the umbrella end [24]. At present, there is no active and effective treatment for infertility caused by incomplete fallopian tube obstruction.

With the extensive development of HyCoSy in clinical practice, infertile patients with incomplete tubal obstruction are screened out in large numbers, and the mechanical effects produced during the process can play a physical dredging role for slight tubal adhesions, which improve postoperative pregnancy rate [2, 25]. Although the rapid flushing of contrast agent can play a role in the physical dredging of mild tubal adhesions, the contrast agent cannot treat tubal mucosal interstitial edema or dissolve adhesions. Anti-inflammatory and anti-adhesion drugs injected into the partially blocked tubal lumen after salpingography can help restore tubal peristalsis and tubal recovery. Of these drugs, dexamethasone belongs to the glucocorticoid class (which is anti-inflammatory), reducing congestion and edema and relieving adhesion [26, 27]. Gentamicin is an aminoglycoside with broad antimicrobial actions and good bactericidal effects [28]. Chymotrypsin is a proteolytic enzyme that lyses proteins and eliminates pus and necrotic tissue [17]. The combination of the 3 drugs can reduce the slight adhesions and strictures of the fallopian tube at higher drug concentrations, promote the dissolution of mild adhesions and eliminate local edema at the lesion site, and relieve the pathologic changes at the site of incomplete obstruction of the fallopian tube to achieve the goal of tubal recanalization.

Our results showed that tubal patency was effectively improved after 4D-HyCoSy combined with salpingitis drug treatment. The cumulative intrauterine pregnancy rate at 6 months after surgery was 26.8%, significantly higher than that of the control group (14.4%; *p* < 0.05) (Table 3). In our study on the correlation between hysterosalpingography and pregnancy showed that the pregnancy rate at 6 months after hysterosalpingography was 19.44% [25], and Emilio Giugliano et al. ‘s study on the therapeutic effect of hysterosalpingography showed that the accumulated pregnancy rate at 6 months after surgery was 22.2% [2], both of which are slightly lower than the results of this study.

**Table 3:** Comparison of intrauterine pregnancy between 2 groups

Group	n	pregnancy		P
		Yes	No	
E-group	82	22 (26.8)^*^	60 (73.2)	0.032
C-group	111	16 (14.4)	95 (85.6)	

Compared with C-group, ^*^P<0.05

In this study, one tubal pregnancy existed in both the experimental and control groups, and the incidence of ectopic pregnancy was 1.2% (experimental group) and 0.9% (control group). The estimated prevalence of ectopic pregnancy reported by Hendriks is 1–2% [29]; these data were comparable, suggesting that for patients with incomplete tubal obstruction, the injection of salpingitis drugs did not increase the risk of ectopic pregnancy.

Eighty-two cases were treated with hysterosalpingography and drug infusion mixture. Among them, 1.2% developed mild vagus nerve reaction and 1.2% developed severe vagus nerve reaction, digital pain score was 2.27, postoperative vaginal bleeding was minor, and no serious complications occurred. Compared with the control group, there was no statistically significant difference in pain and postoperative complications. In a study on the safety, tolerability and pregnancy rate of 660 cases of CEUS reported by Savelli [8], mild vasovagal reaction was 4.1%, severe vasovagal reaction was 0.8%, and digital pain score was 2.7. The incidence of vagal responses and pain scores were similar to this study. Therefore, it can be concluded that continued infusion of salpingitis treatment drugs after 4D-HyCoSy is safe and tolerable.

Our study showed that for patients with incomplete fallopian tube obstruction, adding inflammatory medications after 4D-HyCoSy had a positive effect on postoperative pregnancy rates.

However, the present study have some limitations. Firstly,the data we used were already collected because of our retrospective study, but a prospective trial is necessary to provide additional prognostic factors. Secondly,we could not determine whether the pregnancy is caused by incomplete tubal obstruction without monitoring ovulation. Finally,a large sample study would allow us to strengthen the observed association instead of small sample size in this study.

## Conclusions

We observed that for infertile patients with incomplete obstruction of at least one fallopian tube as diagnosed by 4D-HyCoSy, salpingitis-treatment drugs effectively improved the pregnancy rate postoperatively, with high effectiveness and safety. This regimen is thus worthy of further investigation and promotion in the future.

## Data Availability

The datasets generated and/or analyzed during the current research are not publicly available as individual privacy could be compromised but are available from the corresponding author on reasonable request.

## Abbreviations

4D-HyCoSy: Four-dimensional hysterosalpingo-contrast-sonography
HyCoSy: Hysterosalpingo-contrast-sonography
VAS: Visual analogue scale
TVS: Transvaginal sonography
E-Group: Experimental group
C-group: Control group
CEUS: Contrast enhanced ultrasound
